# A new subtype of Lynch syndrome associated with *MSH2* c.354T>A (p. Y118*) identified in a Chinese family: case report and literature review

**DOI:** 10.3389/fgene.2024.1440179

**Published:** 2024-10-07

**Authors:** Lan Zhong, Wenxiang Wang, Yuanqiong Duan, Liang Song, Zhanghuan Li, Kaixuan Yang, Qintong Li, Rutie Yin

**Affiliations:** ^1^ Department of Obstetrics and Gynecology, West China Second University Hospital, Sichuan University, Chengdu, Sichuan, China; ^2^ Key Laboratory of Birth Defects and Related Diseases of Women and Children, Ministry of Education, West China Second University Hospital, Chengdu, Sichuan, China; ^3^ Department of Gynecologic Oncology, Central Hospital of Xinxiang, The Fourth Affiliated Hospital of Xinxiang Medical University, Xinxiang, Henan, China; ^4^ Department of Pathology, West China Second University Hospital, Sichuan University, Chengdu, Sichuan, China

**Keywords:** Lynch syndrome, endometrial carcinoma, *MSH2*, nonsense mutation, case report

## Abstract

**Background:**

Lynch syndrome (LS) is an autosomal dominant inherited disorder caused by mutations in mismatch repair genes. Genetic counseling is crucial for the prevention and treatment of LS, as individuals with these mutations have an increased lifetime risk of developing multiple cancers. MutS Homolog 2 (*MSH2*) is a protein-coding gene that plays a key role in LS. A significant number of LS cases are linked to harmful heterozygous mutations in the *MSH2* gene.

**Case Presentation:**

The proband was a 50-year-old endometrial dedifferentiated carcinoma patient with a dMMR/MSI-H tumor negative for *MSH2*/*MSH6* expression by immunohistochemistry. Genetic counseling and tumor gene testing were conducted using next-generation sequencing (NGS) technology, which revealed a previously unknown germline *MSH2* gene nonsense mutation NM_000251.2:exon2.354T>A (p.Y118*), leading to a diagnosis of LS. Further analysis of this variant in five family members of the patient confirmed its presence in all individuals, with one family member being diagnosed with colorectal cancer (CRC) at the age of 43. The proband received postoperative chemoradiotherapy and achieved a disease-free survival of 2 years, with ongoing follow-up.

**Conclusion:**

This study provides evidence that the *MSH2* nonsense mutation c.354T>A is a highly likely pathogenic mutation and is responsible for typical LS-associated endometrial carcinoma. It emphasizes the importance of genetic counseling for proband family members to facilitate early diagnosis of LS-related carcinoma.

## 1 Introduction

Lynch syndrome (LS) is an autosomal dominant inherited disorder associated with germline mutations in mismatch repair (MMR) genes, including *MLH1*, *MSH2*, *MSH6*, *PMS2*, and *EPCAM* (Zhao et al., 2022). The MMR system corrects base substitution and insertion–deletion mismatches during DNA replication, leading to microsatellite instability (MSI) (Riedinger et al., 2024). The loss of MMR function is detrimental to genome integrity and sets the stage for cancer development. Clinically, MSI testing—performed by polymerase chain reaction (PCR) analysis—and/or immunohistochemistry (IHC) staining is regularly used in colorectal cancer (CRC) to check MMR deficiency (MMR-D). Because of this, MSI high (MSI-H) and MMR-D are the main genetic signs of LS-related tumors (Fanale et al., 2022). Apart from the increased risk of CRC, LS patients are also susceptible to other primary tumors affecting organs such as the endometrium, ovary, stomach, small intestine, hepatobiliary system, urologic tract, and skin. Women with LS have an approximately 40%–60% chance of presenting a sentinel endometrial carcinoma (EC), which is the most common extraintestinal sentinel cancer in LS patients (Lu et al., 2005; Tafe et al., 2014). Patients with LS-related EC have a 25% estimated risk of developing a second malignancy within 10 years and a 50% risk within 15 years after the initial EC diagnosis (Lynch et al., 1977). Therefore, it is crucial for gynecological oncologists to identify LS among patients with EC.

The prognosis for Lynch syndrome treatment heavily relies on early diagnosis. The screening criteria for identifying Lynch syndrome-associated endometrial carcinoma (LS-EC) have gradually improved. The Amsterdam Ⅱ and Bethesda Guideline, which are based on family history, are widely used clinical criteria to screen for LS in CRC. Extrapolation of both tools to EC has shown a specificity of 61% and 49%, respectively (Syngal et al., 2000). However, universal screening for MMR is recommended for all women diagnosed with EC to identify those with underlying LS (Dillon et al., 2017; Kahn et al., 2019). The newer prediction tools based on molecular screening methods, including MMR-immunohistochemical staining (IHC), microsatellite instability (MSI) testing, and gene sequencing, are widely used to increase the accuracy of screening for LS (Barnetson et al., 2006; Chen et al., 2006) A combination of molecular methods should be used for screening for LS among EC patients (Crosbie et al., 2019). In addition, high-throughput technologies and computational prediction tools, such as deep mutational scanning or multiplexed assays of variant effect (MAVE) technologies, have been developed for assessing variants of unknown significance in Lynch syndrome (Abildgaard et al., 2023). Mutations in *MSH2* or *MLH1* are the most frequently observed genetic mutations in LS patients, accounting for 40% and 50% of LS cases, respectively. Among LS-EC, the mutation rate for *MSH2* genes is 50%–66%, 24%–40% for *MLH1*, and 10%–13% for *MSH6* (Bonadona et al., 2011).

In this study, we identified an EC patient who was diagnosed with LS based on family history and loss of *MSH2* protein in IHC analysis. We conducted a genetic analysis using peripheral blood samples to identify the specific germline mutation. Utilizing second-generation high-throughput sequencing (NGS) technology on the Illumina platform, we analyzed variants, including single-nucleotide variations and small insertions/deletions, in the complete sequences and junction sequences of exons and introns of genes associated with LS in the proband. Through this analysis, we discovered a previously unknown mutation in the *MSH2* gene.

## 2 Case report

Our proband was a 50-year-old Chinese woman (G3P1A2, BMI:23.5 kg/m^2^) who presented with abnormal vaginal bleeding. She had previously undergone fractional curettage through a hysteroscope in another hospital. Pathological results after consultation with pathology experts in our hospital revealed poorly differentiated cancer in endometrium tissue. We performed a radical hysterectomy (Querleu-Morrow, type B), a salpingo-oophorectomy, pelvic lymph node dissection, and a para-aorta lymph node biopsy. Postoperative pathological results revealed that the dedifferentiated carcinoma of the endometrium invaded less than one-half of the uterine muscle, the carcinoma had not invaded the cervical canal, the surgical margins were negative, and no metastatic carcinoma was found in lymph nodes.

**FIGURE 1:**
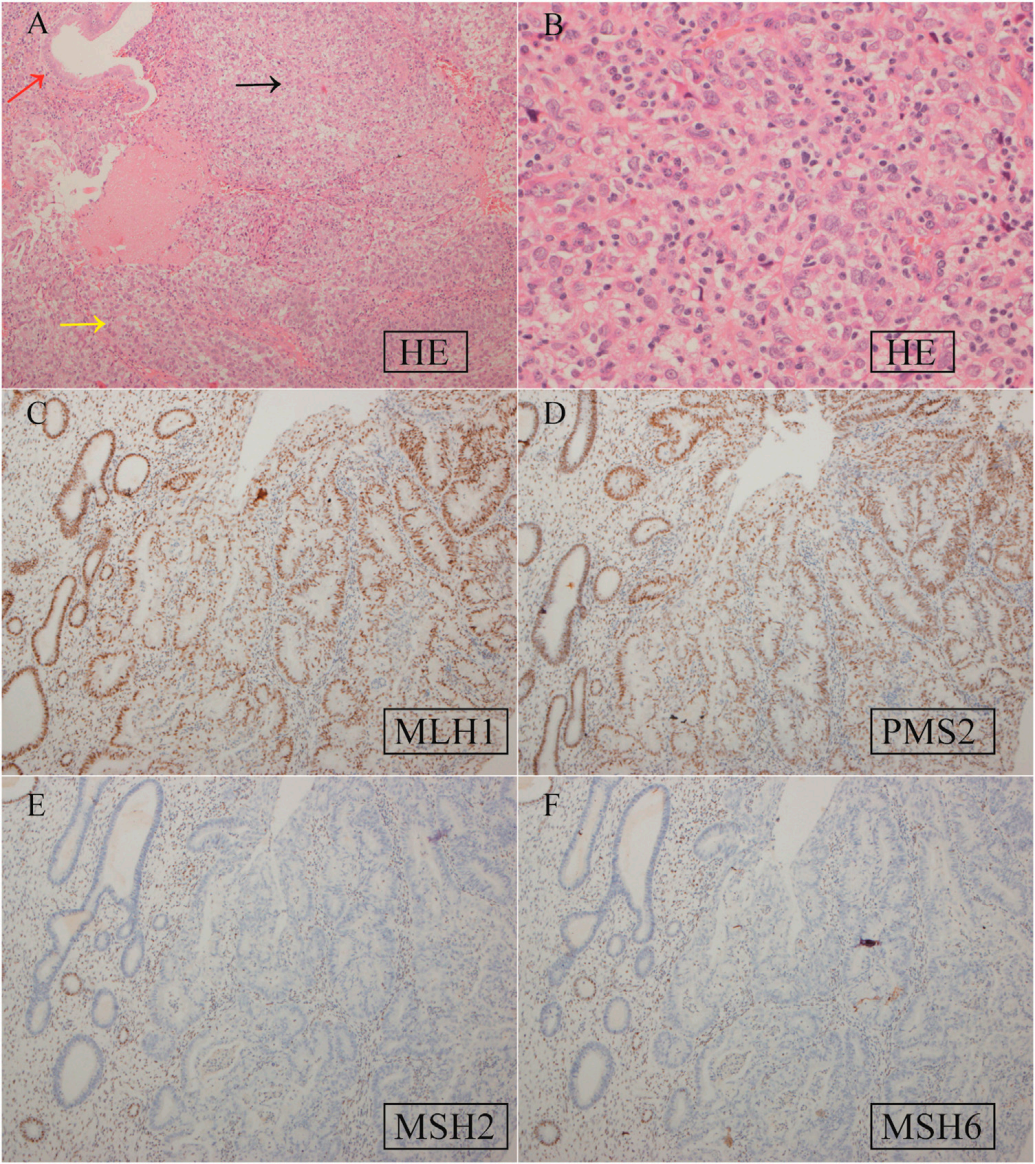
Hematoxylin–eosin (HE) staining **(A, B)** and IHC staining **(C–F)** of EC tissue specimens. **(A)** HE staining. The red, yellow, and black arrows represent normal endometrial glands and well-differentiated and dedifferentiated EC, respectively. Magnification ×200; **(B)** HE staining for dedifferentiated EC. ×400; **(C)** positive *MLH1* expression, ×100; **(D)** positive PMS2 expression, ×100; **(E)** negative MSH2 expression, ×100; **(F)** negative MSH6 expression, ×100.

**FIGURE 2:**
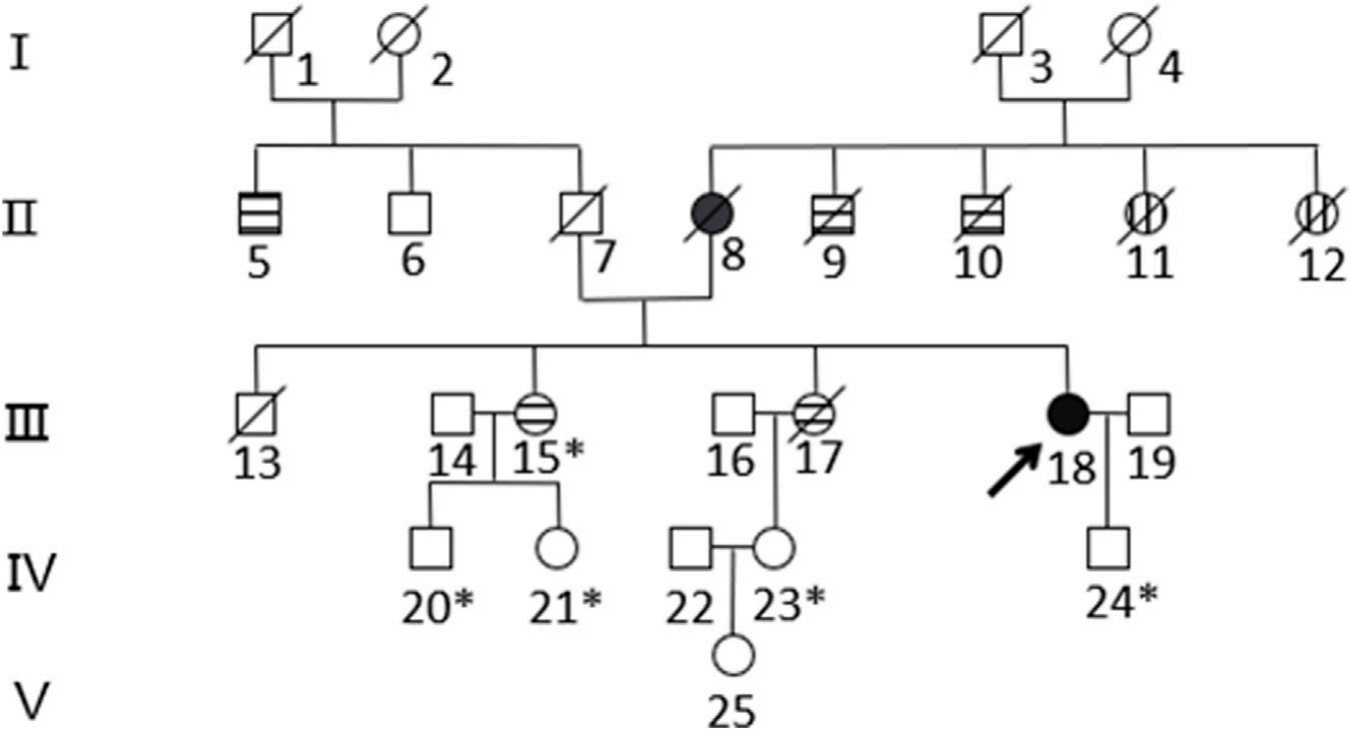
Pedigree of the family with *MSH2* c.354T>A. The arrow (→) indicates the proband. Squares and circles denote men and women, respectively. Roman numbers indicate generations. Black solids represent EC. Patients 8 and 18 suffered from EC at the ages of 57 and 50, respectively. Horizontal stripes represent CRC. Patient 5 suffered rectal cancer at the age of 74. Patients 9 and 10 suffered bowel cancer at the ages of 30 and 70, respectively. Patients 15 and 17 suffered ascending colon cancer at the ages of 43 and 34, respectively. Vertical stripes represent unknown cancer. Patients 11 and 12 suffered unknown cancer in their 30s. A backslash (/) represents people who have died. An asterisk (*) indicates *MSH2* c.354T>A carriers. The remaining family members have not been tested for *MSH2* mutations.

**FIGURE 3:**
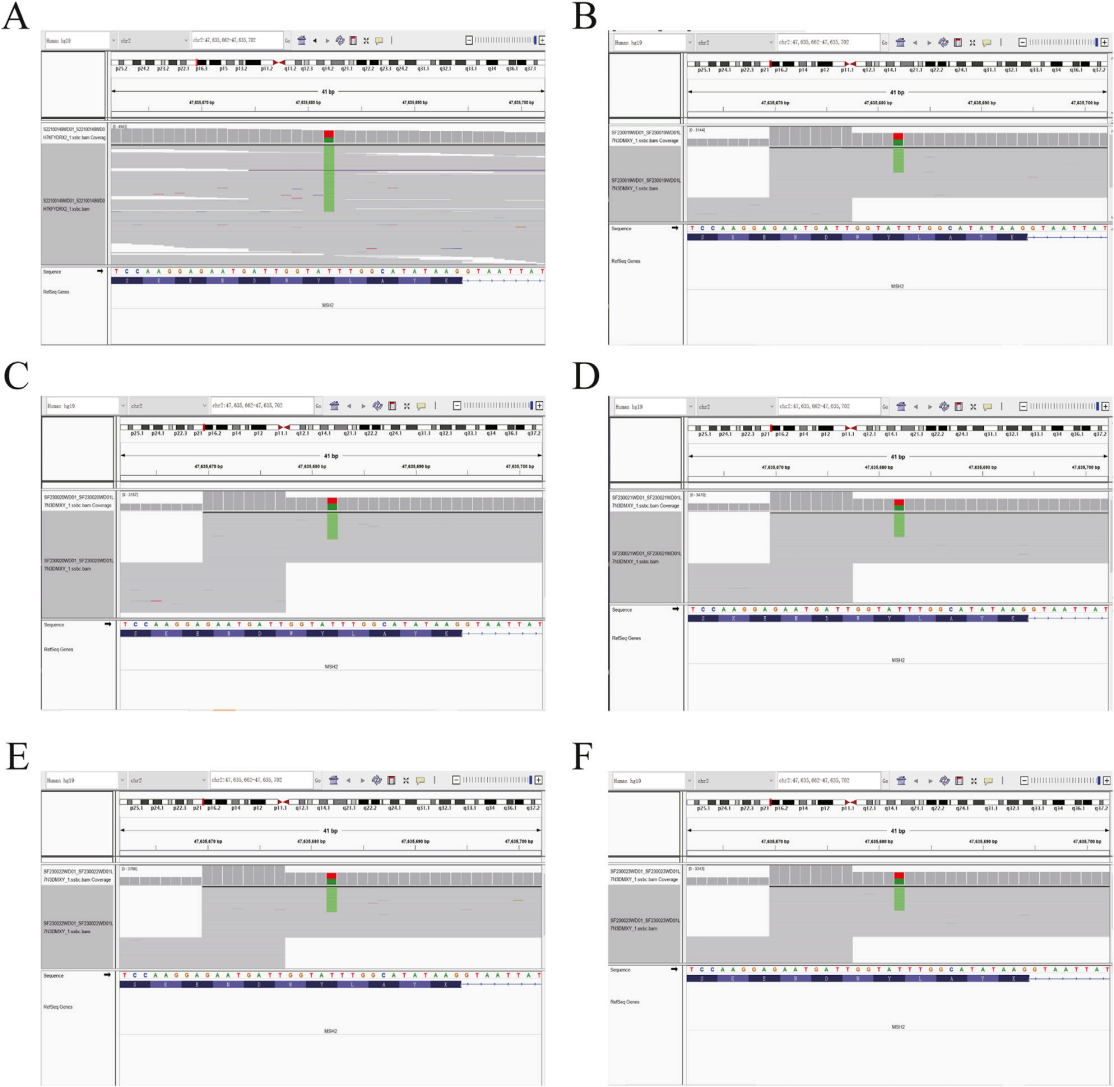
Sequence chromatogram of the proband’s family analyzed by NGS. **(A)** Heterozygous (Het) mutations were observed in the proband. **(B–F)** The same Het mutations were observed in the proband’s five family members. The green stripes show the *MSH2* c.354T>A (p.Y118*) mutation.

Based on the histopathological examination of the sample obtained at surgery, endometrial dedifferentiated carcinoma stage I A was diagnosed. IHC staining demonstrated positive for ER, PR, vimentin, Ki67, MLH1, and PMS2 proteins and negative for MSH2 and MSH6 proteins (Figure 1). Due to a family history of Lynch-related malignancies (Figure 2), the patient met the Amsterdam II criteria and was clinically diagnosed with LS. Therefore, we advised her to undergo genetic counseling and testing. According to The Cancer Genome Atlas (TCGA) molecular subgroups in EC, the molecular classification of this patient was mismatching repair (MMR)-deficient (MMR-d) (Kandoth et al., 2013). Then, in order to identify the germline mutation, we used NGS technology based on the Illumina platform to analyze variants (including single-nucleotide variation and small insertion/deletion) in the complete sequences and the junction sequence of exons and introns of genes related to LS in the proband. Germline testing revealed an NM_000251.2:exon2:c.354T>A (p.Y118*) mutation of the *MSH2* gene, which is a nonsense mutation, resulting in the mutation of the 118th amino acid of the gene coding protein from tyrosine to the termination codon. This mutation is expected to lead to functional damage or inactivation of the protein due to premature protein truncation or nonsense-mediated mRNA decay. NGS was performed to verify the mutation. The functional and clinical significance of this mutation has not been reported in the literature. Genetic analyses of this variant were conducted on five members of this pedigree (Figure 3). All of them were positive for the same *MSH2* gene mutation. Among them, one member (III-15) was phenotypically affected with LS and had been diagnosed with CRC at the age of 43. Four members (IV20, IV21, IV23, and IV24) had been phenotypically free from LS until the screening (Figure 2).

## 3 Discussion

The proband, a 50-year-old woman, initially presented with endometrial dedifferentiated carcinoma. Her mother had EC, while her two uncles and two sisters had CRC, spanning two generations. One of the proband’s uncles (II-9) and two sisters (III-15, III-17) were diagnosed at ages 43 and 34, respectively, in accordance with the Amsterdam II criteria. This pedigree exhibited typical Lynch syndrome (LS)-related cancers, including colorectal, uterine, and an unknown carcinoma.

Genetic testing of MMR genes has been widely used for LS diagnosis. In this report, we demonstrated that the c.354T>A mutation resulted in a nonsense mutation in the *MSH2* gene. The International Society for Gastrointestinal Hereditary Tumors (InSiGHT) database (http://www.insight-group.org) currently lists 9,061 reported *MSH2* mutations. The most common types are frameshift mutations and nonsense mutations, which lead to truncated proteins, accounting for 49% and 19% of all *MSH2* mutations, respectively (Peltomäki and Vasen, 2004).

The MMR proteins form heterodimers, with *MLH1* pairing with *PMS2* and *MSH2* pairing with *MSH6* (Crosbie et al., 2019). These proteins are unstable in their unpaired state, and while *MLH1* and *MSH2* can form stable heterodimers with other proteins, *PMS2* and *MSH6* can only dimerize with *MLH1* and *MSH2*, respectively. Mutation of *MSH2* usually results in IHC loss of both *MSH2* and *MSH6*. In this study, we confirmed the absence of *MSH2* and *MSH6* expression, indicating that *MSH2* is the primary event, and recommended germline testing for LS-associated mutations. Genetic testing of MMR genes was performed to aid the diagnosis of LS. Based on NGS results, a point mutation was identified in *MSH2* (c.354T>A, p.Y118*), located in exon 2. This mutation is a nonsense mutation, leading to the alteration of the 118th amino acid from tyrosine to a termination codon. It is expected that this mutation will result in functional damage or inactivation of the protein through premature protein truncation or nonsense-mediated mRNA decay.

EC is pathogenetically divisible into type I and type II tumors. Type I is low-grade endometrioid adenocarcinoma, and type II is high-grade endometrioid adenocarcinoma and most non-endometrioid carcinomas (Bokhman, 1983). LS-related ECs often exhibit a more diverse histology, including both endometrioid and non-endometrioid types, such as clear cell carcinoma, endometrioid serous carcinoma, undifferentiated carcinoma, and carcinosarcoma (Broaddus et al., 2006). The *MSH2* gene mutation appears to be more frequently associated with a non-endometrioid histology (Broaddus et al., 2006; Carcangiu et al., 2010). However, *MSH2* loss is relatively rarely reported in endometrial undifferentiated carcinoma. Zhou et al. (2020) reported that one of three dedifferentiated endometrioid carcinomas with neuroendocrine differentiation showed a loss of *MSH2*/*PMS6* expression, which is consistent with our findings. These reports suggest a correlation between *MSH2* loss and endometrial dedifferentiated carcinoma.

Compared with the general population, individuals with LS and MMR gene germline mutations have a significantly higher lifetime risk of developing CRC, EC, and other malignancies such as gastric and ovarian cancers (Bonadona et al., 2011; Engel et al., 2012). It is recommended that interventions to reduce the risk of these cancers in affected family members be implemented. In this particular family, genetic analysis was performed on five members of the pedigree (III15, IV20, IV21, IV23, and IV24), and all of them tested positive for the *MSH2* gene mutation. Among them, individual III-15 developed colorectal cancer at the age of 43, while the other four members (IV20, IV21, IV23, and IV24) have not shown any LS-related symptoms so far, possibly due to their young age (16 years, 24 years, 28 years, and 26 years, respectively). This identified mutation is considered “pathogenic” and is likely responsible for the manifestation of LS. Prompt management strategies are crucial for these individuals, particularly for subject III-15, who had already been diagnosed with colon cancer at the age of 43.

There are currently no standardized protocols for the surveillance of MMR pathogenic variant carriers because there are insufficient data to demonstrate clinical benefits. Many reports suggested that the combination of TVS and endometrial biopsy may enhance the efficacy of surveillance (Gerritzen et al., 2009; Auranen and Joutsiniemi, 2011; Tzortzatos et al., 2015; Gambini et al., 2022). According to National Comprehensive Cancer Network (NCCN) guidelines for uterine neoplasms, a yearly endometrial biopsy is recommended for patients and family members with LS but without EC to assess for cancer (Abu-Rustum et al., 2023). Prophylactic hysterectomy/bilateral salpingo-oophorectomy is recommended after childbearing is complete. A retrospective cohort study by Schmeler et al. found that patients who underwent prophylactic gynecologic surgery had no occurrences of EC, ovarian cancer, or primary peritoneal cancer (Schmeler et al., 2006). However, 33% of the patients in the control group were diagnosed with EC. These findings suggest that risk-reducing surgery is an effective strategy for preventing EC in patients with LS. The timing and options of risk-reducing surgery can be individualized based on childbearing potential, comorbidities, menopause status, family history, and types of germline mutations (Seppälä et al., 2021; Abu-Rustum et al., 2023). The risk of LS-EC varies based on the types of germline pathogenic variants (PVs); *MLH1* or *MSH2* carriers might consider risk-reducing surgery at approximately 35 years when they no longer need fertility (Crosbie et al., 2019). For individuals like subject III-15, who has been diagnosed with CRC at the age of 43 and carries an *MSH2* variant without the need for fertility, risk-reducing surgery is recommended. There is no uniform guideline for other young female *MSH2* variant carriers, such as IV-21 (24 years old) and IV-23 (26 years old), due to limited research on gynecologic surveillance in LS. We recommended that the three carriers should raise awareness of “red flag symptoms,” including abnormal bleeding, weight loss, bloating, change in bowel habits, recurrent urinary symptoms, and abdominal discomfort (Funston et al., 2018). When they have fulfilled their reproductive goals, prophylactic hysterectomy/bilateral salpingo-oophorectomy can be considered.

For the monitoring of CRC, individuals with *MLH1* or *MSH2* germline mutations have a 52%–82% risk of CRC by the time they are 70 years old (Bonadona et al., 2011). A systematic literature review showed that an initial screening colonoscopy is recommended from the age of 20–25 years with reexamination every 1–2 years for carriers of *MLHI* or *MSH2* gene mutations, or 10 years younger than the youngest age of the person diagnosed in the family (Lindor et al., 2006). In this pedigree, the youngest age of CRC diagnosis is 34 years (III-17), so we recommended the six carriers (including the proband) undergo colonoscopy every 1–2 years from the age of 24.

## 4 Conclusion

We report a novel case of LS-related endometrial dedifferentiated carcinoma with dMMR/MSI-H by *MSH2* NM_000251.2:exon2:c. 354T>A (p.Y118*), which is considered a likely pathogenetic mutation. We provided *MSH2*-specific genetic counseling to the proband and five other members of her family and confirmed the *MSH2* c. 354T>A (p.Y118*) mutation. There are no publications of LS bearing this variant in the literature. Together with this observation obtained from an LS pedigree, we conclude that this variant may be a likely pathogenic variant of LS.

## Data Availability

The original contributions presented in the study are included in the article/Supplementary Material; further inquiries can be directed to the corresponding authors.
